# Consumer Perceptions of Pattern Hair Loss: Insights from the South Korea Survey

**DOI:** 10.1055/a-2644-3558

**Published:** 2025-08-29

**Authors:** Jino Kim, Jovian Wan, Olena Sydorchuk, Kyu-Ho Yi

**Affiliations:** 1 New Hair Plastic Surgery Clinic, Seoul, Korea; 2 Medical Research Inc., Wonju, Korea; 3 Unicess Inc., Seoul, Korea; 4 Division in Anatomy and Developmental Biology, Department of Oral Biology, Human Identification Research Institute, BK21 FOUR Project, Yonsei University College of Dentistry, Seoul, Korea

**Keywords:** hair, perception, alopecia

## Abstract

Androgenic alopecia is the most common form of non-scarring hair loss, affecting both men and women with distinct regional patterns. This study aimed to investigate public perceptions, treatment behaviors, and preferences related to hair loss. A nationwide online survey was conducted from April 25 to 29, 2024, involving 2,579 participants. The questionnaire assessed sources of information, selection criteria for treatment providers, preferred treatment methods, and use of medications. Respondents most frequently relied on internet communities and social media for information. Treatment provider selection was influenced primarily by online reviews and word of mouth. Common treatments included over-the-counter products, clinic-based therapies, and oral medications. Age and gender differences significantly influenced treatment patterns and perceptions. The findings highlight the importance of digital platforms in shaping patient behavior and underscore the need for evidence-based education to guide individuals seeking treatment for hair loss.

Hair loss is not only a cosmetic concern but also a significant emotional and psychological burden that affects self-esteem, social confidence, and quality of life. As public interest in hair restoration grows, understanding consumer-driven trends and treatment behaviors has become essential for developing patient-centered approaches in aesthetic and dermatologic care.

In recent years, there has been a growing public and clinical interest in hair loss, driven by heightened aesthetic awareness, increased media exposure, and psychosocial concerns associated with thinning hair. This rising demand has stimulated significant advancements in both medical and procedural treatments for androgenic alopecia. Traditional therapies such as minoxidil and finasteride remain widely used, while newer modalities—including low-level laser therapy, platelet-rich plasma (PRP), stem cell-derived exosomes, and hair follicle transplantation—have expanded the therapeutic landscape. Concurrently, patients are increasingly turning to digital platforms for information, reflecting a shift toward self-directed health management and emphasizing the need for accurate, evidence-based guidance in this domain.

Pattern hair loss, medically termed as androgenic alopecia, represents the most common form of non-scarring alopecia. Androgenic alopecia is characterized by the progressive miniaturization of hair follicles, which leads to a significant reduction in hair thickness and density over time. In men, this type of hair loss typically affects the temporal, frontal, and vertex regions of the scalp. In contrast, in women, it predominantly impacts the frontal and parietal regions. 1 2

In this study, a survey was conducted, involving 2,579 participants, from April 25 to 29, 2024. The survey explored various aspects of hair loss, including the preferred sources of information, criteria for choosing treatment providers, treatment methods, and the use of specific medications.

A survey investigating hair loss treatment preferences was conducted through the “Daedamo” website ( https://daedamo.com/ ) from April 25 to 29, 2024, involving 2,579 participants. The demographic composition of *Daedamo* users—primarily adults concerned with aesthetic appearance—aligns well with the target population of this study. While this targeted sampling approach enhances relevance to real-world aesthetic patients, we acknowledge the potential for selection bias, as users of such forums may differ from the general population in terms of health-seeking behavior and treatment awareness. The online survey, organized by Daedamo and utilizing Naver Inc.'s URL format, gathered information through six key sections:

- Participant information: Response timing and consent collection
- Hair loss experience: Information sources and clinic selection criteria
- Treatment methods: Types of treatments used and age of initiation
- Specific treatments: Usage of finasteride and other medications
- Treatment awareness: Knowledge and willingness to use topical finasteride
- Monthly expenditure: Treatment costs

The survey was open to all members without filtering and was designed to understand consumer attitudes, behaviors, and satisfaction levels regarding hair loss treatments. Authors and companies involved in the survey declared potential conflicts of interest.

Results showed that 39% of respondents consider community forums their most trusted information source, followed by blogs (26%) and YouTube (17%; Table 1 ). In selecting treatment providers, 36% favored specialized clinics, while 29% prioritized clinics known for effective treatments. Topical treatments were the most popular method (26%), followed by supplements and functional foods (21%). The survey revealed the profound psychological impact of hair loss and the critical role of community forums and blogs as trusted sources of support.

**Table 1:** Public perception and behavior regarding hair loss treatment

Category	Subcategory	Percentage
Sources of information	Community	39%
	Blogs	26%
	YouTube	17%
	Social media (Instagram, Facebook, etc.)	10%
	Google	7%
	Others	1%
Clinic selection criteria	Specialist in hair loss	36%
	Clinic exclusively treating hair loss	29%
	Local dermatology clinic	21%
	Nearby clinic	10%
	Others	3%
Age at start of treatment	20s	27%
	30s	43%
	40s	22%
	50s	7%
Monthly expenditure	30,000 KRW	27%
	50,000 KRW	31%
	100,000 KRW	21%
	150,000 KRW	9%
	200,000 KRW	8%
	≥300,000 KRW	4%

We performed a cross-sectional analysis of the pattern hair loss perception of consumers. The survey results highlight the complex and multifaceted nature of hair loss and its significant impact on consumers. The psychological effects of hair loss are profound, affecting individuals' self-esteem and social interactions. This underscores the importance of addressing hair loss not just as a cosmetic issue but as a significant factor in mental health. 2 Also, the survey reveals that individuals seeking hair loss treatments rely heavily on community forums, blogs, and YouTube for information, indicating a preference for peer support and detailed personal reviews. Specialized clinics and proven treatment efficacy are primary factors in choosing providers, while topical treatments and dietary supplements are the most common treatment methods. There is significant interest in natural remedies and holistic approaches. Most respondents begin treatment in their 30s, and a majority use finasteride despite concerns about side effects, highlighting the need for better education on medication risks. Expenditure on treatments varies widely, reflecting different financial capacities. The study underscores the importance of patient involvement in treatment decisions and the need for enhanced communication and education regarding hair loss treatments ( Table 2 ). 3 4

**Table 2:** Treatment methods, medication use, and awareness

	Subcategory	Percentage
Hair loss treatment methods	Shampoo or tonic for hair loss	26%
	Dietary supplements or food	21%
	Over-the-counter products	13%
	Consultation with hair loss specialist	10%
	Prescribed medication	9%
	Treatment at hair loss clinic	8%
	Herbal medication	3%
	Other methods (e.g., light therapy)	3%
Finasteride use	Users	57%
	Non-users	43%
Other medication use	Minoxidil	27%
	Dutasteride	19%
	Functional foods	13%
	Bimatoprost	10%
	Other medications	5%
	Others	26%
Awareness of finasteride side effects	Aware	33%
	Not aware	67%
Intention to use topical finasteride	Willing to use	71%
	Unwilling to use	29%

The survey provides valuable insights into consumer perceptions and experiences related to hair loss. Understanding these perspectives can guide the development of more effective and satisfying solutions, ultimately improving the quality of life for those affected by hair loss. As the industry progresses, a consumer-centric approach that addresses both the physical and emotional dimensions of hair loss will be crucial in meeting the needs of this population. 5

By addressing the key concerns and preferences highlighted in this survey, stakeholders can better serve those experiencing hair loss, offering treatments that not only improve hair health but also enhance overall well-being.

A key limitation of this study is its reliance on self-reported data from an online platform, which may introduce selection bias; future research should incorporate clinically verified assessments and more diverse recruitment methods to enhance generalizability. Additionally, the absence of age and gender data restricts subgroup analyses and further limits the applicability of the findings across different demographic groups.
